# Predicting Admissions From a Paediatric Emergency Department – Protocol for Developing and Validating a Low-Dimensional Machine Learning Prediction Model

**DOI:** 10.3389/fdata.2021.643558

**Published:** 2021-04-16

**Authors:** Fiona Leonard, John Gilligan, Michael J. Barrett

**Affiliations:** ^1^Business Intelligence Unit, Children's Health Ireland at Crumlin, Dublin, Ireland; ^2^School of Computer Science, Technological University Dublin, Dublin, Ireland; ^3^Department of Emergency Medicine, Children's Health Ireland at Crumlin, Dublin, Ireland; ^4^School of Medicine, University College Dublin, Dublin, Ireland

**Keywords:** emergency department, paediatric, prediction, machine learning, admission, protocol

## Abstract

**Introduction:** Patients boarding in the Emergency Department can contribute to overcrowding, leading to longer waiting times and patients leaving without being seen or completing their treatment. The early identification of potential admissions could act as an additional decision support tool to alert clinicians that a patient needs to be reviewed for admission and would also be of benefit to bed managers in advance bed planning for the patient. We aim to create a low-dimensional model predicting admissions early from the paediatric Emergency Department.

**Methods and Analysis:** The methodology Cross Industry Standard Process for Data Mining (CRISP-DM) will be followed. The dataset will comprise of 2 years of data, ~76,000 records. Potential predictors were identified from previous research, comprising of demographics, registration details, triage assessment, hospital usage and past medical history. Fifteen models will be developed comprised of 3 machine learning algorithms (Logistic regression, naïve Bayes and gradient boosting machine) and 5 sampling methods, 4 of which are aimed at addressing class imbalance (undersampling, oversampling, and synthetic oversampling techniques). The variables of importance will then be identified from the optimal model (selected based on the highest Area under the curve) and used to develop an additional low-dimensional model for deployment.

**Discussion:** A low-dimensional model comprised of routinely collected data, captured up to post triage assessment would benefit many hospitals without data rich platforms for the development of models with a high number of predictors. Novel to the planned study is the use of data from the Republic of Ireland and the application of sampling techniques aimed at improving model performance impacted by an imbalance between admissions and discharges in the outcome variable.

## Introduction

### Background and Previous Studies

Predicting admissions early in the patient's journey through the paediatric Emergency Department (ED) has potential to improve the patient flow system through both the ED and hospital. One of the influential factors contributing to overcrowding in the paediatric ED is the presence of patients boarding in the treatment area that require admission but cannot leave the ED due to lack of bed capacity in the hospital (Sinclair, 2007). As the volume of patients arriving increases, space, resources, and clinical needs may become an issue as a result of patients boarding in the treatment area, increasing the waiting time for other patients in the waiting room (Chan et al., 2017) and can cause less acute patients to leave without being seen or before the completion of their treatment (Timm et al., 2008; Chan et al., 2017). Early admission prediction would provide advance notice to both ED clinicians and bed managers facilitating decision support and bed planning.

The benefit of using machine learning algorithms to predict admissions was realised in some of the first studies that compared clinical judgement to that of machine learning algorithms (Peck et al., 2012), with many researchers acknowledging that clinical judgement alone, at an early stage, is not enough to accurately predict an outcome of admission (Beardsell and Robinson, 2011; Vaghasiya et al., 2014). A review of the literature has revealed many diverse studies proposing a solution to the question of whether admissions can be predicted from the ED using machine learning algorithms. Some that focus on admission prediction for specific cohorts of patients such as acute bronchiolitis (Marlais et al., 2011) and asthma (Gorelick et al., 2008; Goto et al., 2018; Patel et al., 2018), and others investigating the use of natural language processing to extract valuable information from unstructured text (Lucini et al., 2017; Sterling et al., 2019). A few researchers have concentrated on early prediction (Sun et al., 2011; Lucke et al., 2018; Parker et al., 2019) or progressive time approaches, adding extra information to the model as the patient moves through the ED (Barak-Corren et al., 2017b). There have also been comparisons made between the different machine learning algorithms, with many outperforming the traditional logistic regression classifier (Graham et al., 2018; Goto et al., 2019). The development of tools using minimal predictors to calculate risk of admission scores in some studies (Cameron et al., 2015; Dinh et al., 2016) has underlined the importance of identifying strong predictors for model development.

A review of 26 studies that looked at predicting admissions from the ED provides valuable insight into the types and significance of predictors used (Table 1). The most frequently used predictors were age, sex, triage category, presenting complaint/symptoms, and arrival mode. Apart from sex these were also reported as some of the most influential for predicting admission, particularly at an early stage. To further increase model performance numerous researchers included significant predictors such as vitals (LaMantia et al., 2010; Goto et al., 2019), pain scores (Barak-Corren et al., 2017b), anthropometrics (Barak-Corren et al., 2017a; Patel et al., 2018), medication (Barak-Corren et al., 2017a,b), radiology (Golmohammadi, 2016), and laboratory (Kim et al., 2014; Barak-Corren et al., 2017b) tests ordered. For one paediatric study that created models after 0, 10, 30 and 60 min, the inclusion of these types of predictors resulted in an Area Under the Curve (AUC) of 0.789 for 0 min up to an outstanding discrimination value of 0.913 at 60 min upon evaluation (Barak-Corren et al., 2017a).

**Table 1:** Top 15 variables considered for inclusion across 26 studies.

**References**	**Study type**	**Age**	**Sex**	**Triage/Acuity**	**Complaint/Symptoms**	**Arrival mode**	**Arrival time**	**Weekday**	**Vitals**	**Previous visits/Re-attend**	**Previous admission**	**Laboratory tests**	**Race/Ethnicity**	**Radiology tests/ECG**	**Referral source/Residence type**	**Chronic condition/Co-morbidity**
Araz et al. (2019)	Adult	++	o	++	o	++	o	o								
Barak-Corren et al. (2017b)	Mixed	+	o	+	+	+		o	o	+	+	o	+	o		
Barak-Corren et al. (2017a)	Paediatric	+	o	+	+		+	+	+	+	+	+				o
Cameron et al. (2015)	Adult	++	o	++		++	o	o		o	++				++	
Considine et al. (2011)	Adult	++	o	o	+	+			++							
Dinh et al. (2016)	Adult	++	o	++	++	++	++			o	++		+		+	
Golmohammadi (2016)	Mixed	++	++		++	++	++	o						++		
Gorelick et al. (2008)	Paediatric	o	o		o				++	o	+					
Goto et al. (2019)	Paediatric	++	o		++	++			++	o					o	o
Goto et al. (2018)	Adult	++	o		o	++			++							++
Graham et al. (2018)	Mixed	++	+	++		++	+	+			++					
Hong et al. (2018)	Not stated	++	++	++	o	+	o	o	+	++	++	o	++	o		o
Kim et al. (2014)	Adult	++	+	++	++	++	++	++				++			++	
Kraaijvanger et al. (2018)	Mixed	++	o	++	++	++		o				+		+	++	+
LaMantia et al. (2010)	Adult	++	o	++	++				++				o			o
Leegon et al. (2005)	Adult	o		o	o	o	o					o		o		
Leegon et al. (2006)	Paediatric	o	o	o	o	o						o		o		
Li et al. (2009)	Not stated	o	o	o	++		o									
Lucke et al. (2018)	Adult	+	+	++	++	++			++	o		+				
Marlais et al. (2011)	Paediatric	++			o				++	o						
Parker et al. (2019)	Adult	++	+	++	+	++	+	+		+			+			
Patel et al. (2018)	Paediatric	++	o	++					++				o			
Peck et al. (2012)	Adult	++		+	++	++										
Peck et al. (2013)	Adult	++		++	++	++										
Rendell et al. (2019)	Adult	++	o	++	++	++	o	o		o	++		o		o	
Sun et al. (2011)	Mixed	++	+	++		++				+	+		+			+

*o considered or added to prediction model*.

*+ reported as significant in bivariant tests, if carried out (p < 0.001)*.

*++ highlighted in respective study as the top variables of importance or strongest predictors in final models*.

The key design elements from each of the 26 studies were also analysed, 54% originated in the US followed by Australia at 15%. Sample sizes vary, from 321 case records in research focused on COPD (Considine et al., 2011) to 1,721,294 case records in a state-wide study carried out in Australia (Rendell et al., 2019). For the most, data is partitioned into 2, train and test/validation, 16 studies followed this method compared to 5 who partitioned into 3 separate samples (train, test, and validation). Lower rates of admission can be seen in paediatric research at 4.5% (Goto et al., 2019) compared to 65.4% in a study for older patients (75+ years) (LaMantia et al., 2010). One of the highest rates of admission was observed in a COPD study at 77.3% (Considine et al., 2011). The definition of “admission” in the outcome variable also sees some differences, the majority of papers define this as hospital admission, but some have additional criteria. These include planning, assessment and short stay units (Gorelick et al., 2008; Considine et al., 2011; Dinh et al., 2016; Rendell et al., 2019), transfers to another hospital (Goto et al., 2018, 2019; Lucke et al., 2018; Rendell et al., 2019), and deaths in the department (Cameron et al., 2015) (Supplementary Appendix 1).

The most common exclusions consist of died at the ED or on arrival, decision to leave the ED, missing data, patients for direct admission or planned re-evaluation, age criteria, and triage categories that may result in quasicomplete separation. Logistic regression is the traditional choice of classifier for this type of research, with 24 out of the 26 studies using it for model development. Many other types of classifiers have been explored, 7 researchers have used neural networks and 6 have developed machine learning algorithms using ensemble methods (Supplementary Appendix 1). When it comes to evaluation and model comparison, 88% reported the AUC followed by specificity and sensitivity documented in 19 papers. As logistic regression is the most used classifier, odds ratios or coefficients were provided in 18 studies. Positive and negative predictive values were also widely reported in over 58% of studies. Comparing the highest AUC results achieved for each study, one of the lowest was reported as 0.73, which related to research undertaken to predict admissions for patients 75 years and over from the ED. The admission rate was 65.4% and used 5 predictors; age, heart rate, diastolic blood pressure, triage and chief complaint (LaMantia et al., 2010). The highest AUC reported was 0.97, which included 43 parameters and used a progressive time approach, comparing models with data captured within 10 min, 1 and 2 h. Within 1 h, test results were included and physician diagnosis within 2 h. The researchers acknowledged that one of the strongest predictors was full blood work ordered, with 89% of these patients hospitalised (Barak-Corren et al., 2017b; Table 2).

**Table 2:** Most commonly used model evaluation methods and AUC results across the 26 studies.

**References**	**AUC**	**Specificity**	**Sensitivity**	**Odds ratios/Coefficients**	**PPV**	**NPV**	**Accuracy**	**PLR**	**NLR**	**Distinct AUC results**
Araz et al. (2019)	X			X	X		X			0.77, 0.79, 0.81, 0.83, 0.84, 0.86
Barak-Corren et al. (2017b)	X	X	X	X	X	X	X			0.79, 0.87, 0.91
Barak-Corren et al. (2017a)	X	X	X		X	X	X	X	X	0.82, 0.83, 0.86, 0.96, 0.97
Cameron et al. (2015)	X	X	X	X	X	X				0.88
Considine et al. (2011)				X						
Dinh et al. (2016)	X	X	X	X	X	X	X			0.82
Golmohammadi (2016)		X	X	X			X			
Gorelick et al. (2008)	X	X	X	X				X	X	0.92
Goto et al. (2019)	X	X	X	X	X	X		X	X	0.78, 0.80
Goto et al. (2018)	X	X	X		X	X				0.82, 0.83
Graham et al. (2018)	X	X	X	X			X			0.82, 0.85, 0.86
Hong et al. (2018)	X	X	X		X	X				0.86, 0.87, 0.91, 0.92
Kim et al. (2014)	X			X			X			0.68, 0.75, 0.77, 0.80, 0.82, 0.84
Kraaijvanger et al. (2018)	X	X	X	X	X	X				0.76, 0.84, 0.87
LaMantia et al. (2010)	X			X						0.73
Leegon et al. (2005)	X	X	X		X	X				0.89
Leegon et al. (2006)	X	X	X		X	X				0.90, 0.91
Li et al. (2009)		X	X				X			
Lucke et al. (2018)	X	X	X	X	X	X		X	X	0.77, 0.86
Marlais et al. (2011)	X	X	X	X	X	X				0.81
Parker et al. (2019)	X	X	X	X	X	X				0.83
Patel et al. (2018)	X	X	X							0.72, 0.82, 0.83, 0.84
Peck et al. (2012)	X			X						0.84, 0.89
Peck et al. (2013)	X			X						0.80, 0.82, 0.86, 0.89
Rendell et al. (2019)	X						X			0.82, 0.83 (highest AUC out of 2 sets of models)
Sun et al. (2011)	X	X	X	X	X	X				0.85
Total	23	19	19	18	15	14	9	4	4	

*AUC, Area under the curve; PPV, Positive predictive value; NPV, Negative predictive value; PLR, Positive likelihood ratio; NLR, Negative likelihood ratio*.

Evident in many paediatric studies is the lower admission rate (Goto et al., 2019) which may impact model performance resulting in low positive predictive values or sensitivity. Class imbalance occurs when the class of interest (admission) is represented by a lower number of observations compared to the majority class (discharge). The application of sampling techniques such as undersampling and oversampling which will be explored in the proposed study, can improve model performance by changing the data distribution in the training set, providing a more evenly distributed balance between the 2 classes (Han and Kamber, 2012).

Our proposed study will look at the creation of a low-dimensional model which would be of benefit to many countries that do not have data rich platforms when developing and deploying predictive models. The World Health Organisation reports a 50% adoption rate of national Electronic Healthcare Record systems, which capture this data in the upper to middle income and high income bracket countries in comparison to the lower to middle income and lower income bracket countries were a much lower uptake is reported. In addition legislation governing use of Electronic Health Records follows the same pattern (World Health Organisation, 2019).

### Research Aim

The aim of this research is to develop and validate a low-dimensional machine learning model that can predict admissions early from a paediatric ED. To our knowledge, this will be the first study carried out using data from a paediatric hospital in the Republic of Ireland. This will be achieved by creating 15 models derived from 5 different sampling strategies and 3 machine learning algorithms. These models will be trained on predictors identified in previous research, comprised of routinely collected data entered up to the post-triage process. The variables of importance will be identified from the model with the highest AUC and these predictors will then be used to create an additional low-dimensional model. A low-dimensional model that uses commonly collected data has the potential to generalise better in hospital environments that have a lower level of information technology maturity.

## Methods and Analysis

The reporting guidelines set out by Transparent Reporting of a Multivariable Prediction Model for Individual Prognosis or Diagnosis (TRIPOD) (Collins et al., 2015) will be followed.

### Study Design

This study will follow the data mining methodology, Cross Industry Standard Process for Data Mining (CRISP-DM) consisting of 6 key phases (Wirth and Hipp, 2000); business understanding, data understanding, data preparation, modelling, evaluation, and deployment. Data extraction and transformation will be performed using Microsoft SQL Server Management Studio, with subsequent data preparation, modelling, and evaluation to be carried out using R Studio Version 1.1.456. From 3 different machine learning algorithms and 5 sampling techniques, 15 models will be developed. The best performing model will be selected based on the highest AUC, from which the variables of importance will also be derived and used to create a further low-dimensional model.

### Data Sources and Sample Size

Data will be extracted from 3 separate information systems and will use the patient's healthcare record number as the common link. Most of the data will be retrieved from the ED information system, with the patient administration system and inpatient enquiry system providing hospital admission usage and medical history data. The study sample will consist of 2 years of data from 2017 to 2018, providing a good of representation of seasonal changes and the unique values within each variable. Based on the average attendance per year, the sample size will be ~76,000.

### Study Participants and Exclusion Criteria

All attendances to one acute paediatric ED in the Republic of Ireland will be included. Visits will be excluded for the following:

1. Patients over 18 years of age.
2. Visits where the patient left without being seen or left before completion of treatment.
3. Patients returning for direct day case surgical management.

Missing data will be analysed, listwise deletion will be performed depending on the percentage of missing values and whether those values are missing at random. Otherwise the most appropriate principled method to handle missing data will be applied. These methods may include multiple imputation, expectation-maximum algorithm or full information maximum likelihood (Dong and Peng, 2013).

### Outcome and Predictors

The outcome to be predicted is “admission” or “discharge.” Patient visits with a discharge outcome of admission, transferred to another hospital for admission (Goto et al., 2018; Lucke et al., 2018) and died in department (Cameron et al., 2015) will be grouped into the category of “admission,” all other visit discharge outcomes will be defined as “discharge.”

Based on a review of the literature the following predictors, comprised of both numerical and categorical data types will be included in the study.

#### Demographics

Age, sex, and distance travelled. Distance travelled will be measured in kilometres and will be calculated from the patient's home address to the hospital site.

#### Registration Details

Arrival mode, referral source, registration date and time (split into weekday, month, and time), re-attendance within 7 days, presenting complaint and infection control alert.

#### Triage Assessment

Triage category, first ED location and first clinician type assigned to. The Irish Children's Triage System is used to assign triage categories and in order to prevent quasicomplete separation occurring (Kraaijvanger et al., 2018), whereby near perfect prediction of “admission” is obtained for triage 1 and “discharge” for triage 5, triage will be grouped into 1–2, 3, and 4–5.

#### Hospital Usage

Previous visits to the ED (within previous year) and previous admissions (within previous 7 days, 30 days, 1 year, and all previous admissions).

#### Past Medical History

Eleven binary predictors will be created based on paediatric complex chronic conditions as detailed in a study by Feudtner et al. (2014), using the ICD10 diagnosis codes from the patients previous admissions. Based on the previous 3 years admissions, diagnostic related groups specific to blood immunology and digestive system groups will be created, representing specific cohorts of patients attending this facility.

During the data understanding phase descriptive statistics will be produced consisting of stacked bar charts for categorical data and shape, location, and dispersion for continuous variables. The results will be analysed to inform any data quality issues to be addressed and feature engineering tasks to be performed on each predictor. Statistical data preparation tasks will include bivariant tests to assess independence of each predictor with respect to the outcome variable, Pearson's chi square test for categorical and *t*-tests for continuous variables. To identify any possible multicollinearity issues the variance inflation factor will be produced and reviewed for each predictor. These data preparation tasks will further inform any potential variable exclusion from the final dataset.

### Data Analysis Plan

Using random sampling the finalised dataset will be split into 70% train and 30% test. The training set to be used during the model learning process and the test set for validation and evaluation, using unseen samples to provide an unbiased evaluation. A flow diagram will be produced detailing the exact breakdown of the dataset into excluded and included attendances. Under excluded attendances, each individual exclusion criteria will be represented. Under the included attendances, a breakdown between the training and test sets will be shown and will be further broken down into the distribution of “admission” and “discharge” attendances for the outcome variable. This visual representation will show the exact number of samples and the percentage distribution.

As standard machine learning algorithms assume a balanced training set (López et al., 2013), 4 additional sampling techniques will be considered for application to the training set to address the class imbalance. These sampling techniques will be implemented before the learning step and therefore have the potential to increase model performance. The 5 training sets will consist of:

1. **Reference:** The original untouched training set will be used as the reference.
2. **Undersampling**: Random undersampling will decrease the number of observations in the majority class “discharge” until there is an even distribution of observations for “admission” and “discharge.”
3. **Oversampling**: Random oversampling will resample the minority class “admission,” until both the “admission” and “discharge” classes have an even distribution of observations.
4. **Synthetic Minority Oversampling Technique** (Chawla et al., 2002) **(SMOTE)**: SMOTE will under-sample the majority class “discharge” and will then use a k nearest neighbour approach to synthesise new “admission” observations resulting in an even class distribution in the outcome variable.
5. **Random Oversampling Examples** (Menardi and Torelli, 2014) **(ROSE):** ROSE will use a smoothed bootstrap technique to resample the minority class “admission” until there is an even distribution between the 2 outcome classes.

**Figure 1:**
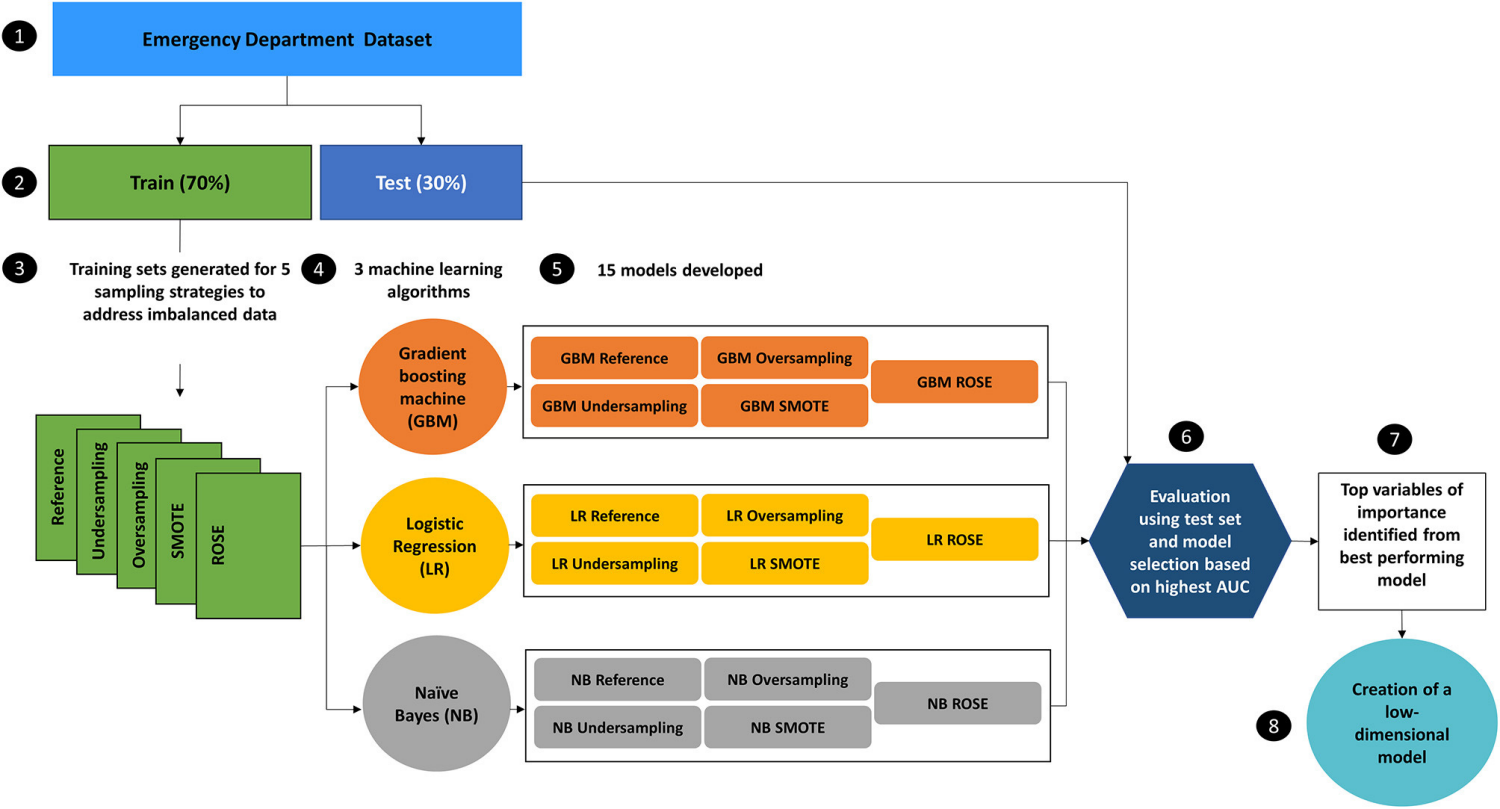
Design of experiment to identify the model with the highest Area Under the Curve (AUC) from 15 models which will be used to obtain the variables of importance for the creation of a low-dimensional model. The reference training set will have no additional sampling technique applied.

Three machine learning algorithms will be used to compare performance across the 5 different training sets, resulting in the development of 15 models (Figure 1). Logistic regression which is the traditional choice of classifier for this field of study will be compared with naïve Bayes and the ensemble method, gradient boosting machine. These machine learning algorithms were selected as they can be used directly with categorical data that has not been encoded. Both logistic regression and naïve Bayes were used extensively in previous studies, with the gradient boosting machine algorithm achieving a higher AUC than other classifiers (Graham et al., 2018; Patel et al., 2018; Goto et al., 2019), therefore providing a good basis for comparison (Supplementary Appendix 1). The optimal tuning parameters for both the naïve bayes and the gradient boosting machine algorithms will be selected by creating a custom tuning grid and using 10-fold cross validation.

The models will be validated and evaluated by applying the test set. Performance will be measured primarily using AUC, with specificity, sensitivity, accuracy, positive prediction value and negative prediction being produced as the secondary measurements. Confidence Intervals at 95% will be generated for each measure. When reporting these measures and to assist comparison, the specificity will be fixed at 90% to evaluate the true impact of applying the different sampling methods for imbalance at a common fixed point.

The variables of importance will be obtained from the model with the highest AUC. The calculation of relative importance of each predictor will differ depending on the machine learning algorithm and will be calculated for the optimal model only. For logistic regression, the odds ratios and regression coefficients will be produced. The a priori and conditional probabilities will be examined for naïve Bayes and the average decrease in mean squared error for the gradient boosting machine will be produced. A low-dimensional model will then be created based on the top variables of importance. The number of dimensions to be included will be determined by assessing the AUC, beginning with the top 10 variables, and reducing the number of variables according to relative importance.

## Discussion

### Sampling Approaches and Low-Dimensional Modelling

The approach taken to address the class imbalance problem in most studies is to apply the technique of threshold moving. One method taken was to adjust the threshold to maximise the specificity for bed mangers which will control the number of false positives and to increase sensitivity for use by clinicians in the ED for decision support (Sun et al., 2011). Other groups fix the specificity at 90% (or over) to increase sensitivity (Leegon et al., 2006; Barak-Corren et al., 2017a) or use statistical approaches like Youden's index to identify the optimal threshold for a balance between sensitivity and specificity (Hong et al., 2018). The technique of threshold moving has no impact on model performance in terms of AUC, it merely adjusts the output threshold so that the rare class (admissions) are easier to classify.

Often standard classification algorithms are biased towards the class representing the majority (discharge) which introduces a higher misclassification rate for the minority (admission) class, the main class of interest (López et al., 2013). To mitigate this, data level approaches applied before the model learning stage, such as sampling techniques including oversampling, undersampling and synthetic versions of oversampling, have proven to be efficient (Santos et al., 2018) and unlike threshold moving, they can potentially increase model performance including AUC. Novel to this type of study, we will investigate the effect of applying these sampling techniques at data level to potentially improve model performance. The yearly admission rate from our paediatric ED is approximately 15%, which confirms an imbalance in the outcome variable that may influence the model's ability to correctly classify the minority class (admission).

We propose creating a low-dimensional machine learning prediction model based on routinely collected data up to the post-triage process. From the literature review, the most common and successful predictors were obtained and used to assess which data could be included in the formation of our dataset. Not all hospital environments are at the same level of information technology maturity and therefore may also have limited data to form these datasets, with many predictors heralded as being significant in previous studies, not available to them. The approach we have taken focuses more on generalisability, by identifying significant predictors to use in a low-dimensional model. A model that will use 10 or less variables based on commonly collected data to make a prediction. In a study by Peck et al. (2013), generalising a model was explored, evident from this study was the low number of predictors included (6 in total), although AUC results were lower than more recent studies (Barak-Corren et al., 2017a; Hong et al., 2018) that included more variables, the study successfully demonstrated how a low-dimensional model could be used across different hospitals.

This low-dimensional model could be deployed for use by ED clinicians as an additional decision support mechanism and would also be useful for bed management to assist advance bed planning. The output of the model could be integrated into Electronic Healthcare Record/ED information systems, displaying the percentage chance of admission of each patient. Several studies have suggested aggregating the raw probabilities to increase the accuracy of the number of beds required (Peck et al., 2012; Cameron et al., 2015) for bed managers, others recommending to display the probability of admission at patient level (Barak-Corren et al., 2017a). It is clear that ED dashboards that are designed to improve situation awareness and decision support (Franklin et al., 2017; Yoo et al., 2018) can also be further enhanced with the inclusion of predictive analytic results.

### Limitations

There are some potential limitations to the planned study. Data will be included from one paediatric ED in the Republic of Ireland, with an expectation of expanding the research in the future to include multiple sites. There may also be limitations based on the data available or local categorisation. The first ED location the patient is assigned to, post triage can only be accurately grouped into “Resus” or “Other.” The presenting complaint uses a local grouping system and the Irish Children's Triage System was used to assign the patient's triage category. This will be a single site study. Some of the predictors that emerge may be due to local context. However, the proposed methodology to obtain the variables of importance is transferable to other settings. It can be used to develop low-dimensional models and it would be valuable to consider a comparative analysis of these in the future.

## Dissemination

The results and subsequent paper will be disseminated in a manuscript to peer-reviewed journals. The finalised raw data used to create the low-dimensional model will be made available on https://figshare.com/.

## Ethics Statement

The studies involving human participants were reviewed and approved by Hospital Research and Ethics Committee of Children's Health Ireland at Crumlin (reference code GEN/693/18). Written informed consent from the participants' legal guardian/next of kin was not required to participate in this study in accordance with the national legislation and the institutional requirements.

## Author Contributions

FL, JG, and MB conceived the study, have made a substantial contribution to this manuscript, give their final approval of the submitted version, and agree to be accountable for all aspects of the work in ensuring that questions related to the accuracy or integrity of any part of the work are appropriately investigated and resolved. FL prepared and finalised the manuscript. All authors contributed to the article and approved the submitted version.

## Conflict of Interest

The authors declare that the research was conducted in the absence of any commercial or financial relationships that could be construed as a potential conflict of interest.
