# Supplementary material for: Predicting Admissions From a Paediatric Emergency Department – Protocol for Developing and Validating a Low-Dimensional Machine Learning Prediction Model
Source: Front Big Data. 2021 Apr 16;4:643558. doi: 10.3389/fdata.2021.643558 (PMC8085432; doi:10.3389/fdata.2021.643558)
Supplement: Supplementary file 1 [file Table_1.docx]

Supplementary Material

# Supplementary Table 1

**Supplementary Appendix 1:** Key design elements for 26 studies on predicting admissions from an Emergency Department

| **Author Group** | **Study Type** | **Sample** | | | **Split** | | | **% Admission Rate** | **Outcome definition of Admission** | **Exclusions** | **Classifiers and Machine Learning Algorithms** |
| --- | --- | --- | --- | --- | --- | --- | --- | --- | --- | --- | --- |
|  |  | **Size** | **Age Inclusion** | **Time Period** | **Train** | **Test** | **Validation** |  |  |  |  |
| Araz et al., (2019) | Teaching hospital, metropolitan-area, US. | 118,005 | Mean in years (SD) 37.42 (23.26) | 2006-2009 | 70% | 15% | 15% | 24.7% | Admission to hospital |  | Logistic regression; Artificial neural network; Support vector machine; Decision tree; Random forest; Extreme gradient boosting methods |
| Barak-Corren et al., (2017b) | Boston Children’s Hospital, US. | 59,033 | Mean (95% CI) in months. Admitted: 103.9 (102.3-105.4) Discharged: 92.93 (92.2-93.7) | 1 July 2014 to 30 June 2015 | 50% |  | 50% | 20.3% | Admission to hospital | Left against medical advice; Died at ED; Left without being seen. | Mixed method, running a logistic regression model on output of a naive Bayes classifier |
| Barak-Corren, et al., (2017a) | Tertiary care hospital in Haifa, Israel. | 80,880 | Range 0-107 years, Mean (Median) 42.8 (39) | January 2011 to January 2012 | 50% | 50% |  | 28.0% | Admitted to inpatient department | Deceased; Other institutes; Unknown outcome; Decided to leave on their own. | Logistic regression; Naïve Bayes; C4.5 classification tree |
| Cameron et al., (2015) | Two Medical Acute Assessment Units, and one Minor Injuries Unit in North Glasgow, UK. | 322,846 | 16 - 90+ years | 21 March 2010 to 20 March 2012 | 67% |  | 33% | Not Stated | Admitted and deaths in department | Under 16 years of age. | Logistic regression |
| Considine et al., (2011) | COPD - major private hospital and four urban district public hospitals in metropolitan Melbourne, Australia. | 321 | Over 18 years | July 2006 to July 2007 | 100% |  |  | 77.3% | Hospital admission (including planning units, medical assessment, and short stay) | Included all acute respiratory illness diagnosis that will exacerbate COPD. | Logistic regression |
| Dinh et al., (2016) | Level 5 or 6 Emergency Department, state-wide New South Wales, Australia. | 1,721,294 | 16+ years | 2013 and 2014 | 50% |  | 50% | 40.7% (Based on training set) | Inpatient admission (including medical assessment and short stay units). | Dead on arrival; Planned representations; Patients who did not wait for triage; Missing or uninterpretable presenting problem field entries. | Logistic regression; Lasso regression |
| Golmohammadi, (2016) | Urban hospital in the Boston area, US. | 10,380 | 1 day - 102 years | January 2012 to August 2012 | 70% | 30% |  | 16.8% |  | Observations with missing data. | Logistic regression; Artificial neural network |
| Gorelick et al., (2008) | Asthma - 2 urban paediatric EDs in US | 1,221 | 24 months - 18 years | 12 months | Data from 1 ED (70%) |  | Second site in a different city (30%) | 41.3% | Inpatient and short stay care | No English or Spanish speaking caretaker; ED revisit for the same condition in the previous 7 days; Other chronic cardiorespiratory disease; Respiratory failure requiring immediate intubation. | Logistic regression |
| Goto et al., (2019) | Nationally representative sample using combined data from the ED section of the National Hospital Ambulatory Medical Care Survey (NHAMCS). 50 US states and the District of Columbia. | 52,037 | ≤ 18 years | 1 January 2007 to 31 December 2015 | 70% | 30% |  | 4.5% | Inpatient Admission and transfer to acute care hospital | Missing triage classification; Dead on ED arrival; Left before being seen or against medical advice; Vital data inconsistencies. | Lasso regression; Random forest; Gradient-boosted decision tree; Deep neural network |
| Goto et al., (2018) | Asthma and COPD exacerbations from National Hospital and Ambulatory Medical Care Survey (NHAMCS). 50 US states and the District of Columbia. | 3,206 | ≥ 18 years | 2007–2015 | 70% | 30% |  | 26.0% | Inpatient Admission and transfer to acute care hospital | Missing predictor variables; Dead on ED arrival; Left before being seen or against medical advice. Included ICD-9-CM diagnoses of Asthma or COPD exacerbation | Logistic regression with lasso regularization; Random forest; Gradient boosted decision tree; Deep neural network |
| Graham et al., (2018) | Two acute hospital EDs in Northern Ireland, UK. | 107,545 | Mean in years (SD) 43.21 (26.2) | 2015 | 80% | 20% |  | 24.7% | Admitted to hospital | Patients attending direct assessment and observation units. | Logistic regression; Decision tree; Gradient boosted machine. |
| Hong et al., (2018) | Represented EDs include a level I trauma centre a community hospital-based department and a suburban, free-standing department in US. | 560,486 | Mean in years (61.6 Admitted and 44.9 discharged) | March 2014 and July 2017 | 80% | 10% | 10% | 29.7% | Patient disposition of admission | Dispositions other than admission or discharge. | Logistic regression; Gradient boosting; Deep neural network |
| Kim et al., (2014) | Tertiary referral urban hospital in Australia | 100,123 | ≥ 18 years | January 2010 and March 2012 |  |  |  | 38.6% | Admission to hospital | Transferred from other hospitals (blood tests usually performed before their transfer). | Logistic regression |
| Kraaijvanger et al., (2018) | EDs of 1 academic and 2 community hospitals in the Netherlands | 3,174 | 0 - 89+ years | Between 10 and 16 January 2011 and between 9 and 15 May 2011 | 40% (1 ED) |  | 44% (1 ED) and 16% (1 ED) | 31.7% (Based on training set) | Admission to hospital | Observations with variables that resulted in quasicomplete separation. | Logistic regression |
| LaMantia et al., (2010) | Tertiary care, Level 1 trauma centre in North Carolina, US. | 5,716 | 75+ years | 2007, June 2008 and December 2008 | 85% |  | 15% | 65.4% | Admission to hospital | Direct admission or inbound hospital transfer that were not registered; Medical evaluation was not carried out in the ED. | Logistic regression |
| Leegon et al., (2005) | Urban Trauma Level I centre, US. | 16,967 | Adults | 10 April 2004 to 31 Aug 2004 | 39.3% (10 April to 30 June 2004) | 16.8% (10 April to 30 June 2004) | 43.9% (1 July to 31 August 2004) | 29.0% | Admission to hospital | Died in the ED; Left without being seen and left against medical advice. | Bayesian network |
| Leegon et al., (2006) | Paediatric Level 1 Trauma centre, US. | 43,077 | Paediatrics | 10 April 2004 to 30 June 2005 | 10 April to 30 June 2004 | 1 July to 31 August 2004 | 1 September 2004 to 30 June 2005 | 15-16% | Admission to hospital | Died in the ED; Left without being seen and left against medical advice; Transferred out to another facility. | Artificial neural network |
| Li et al., (2009) | University Hospital, tertiary care centre, US. | 2,784 |  | January 2008 |  |  |  | 28.0% | Admission to hospital |  | Logistic regression; naïve Bayes; Decision tree; Support vector machine |
| Lucke et al., (2018) | Tertiary-care hospital in the Netherlands | 21,287 | ≥ 18 years | 2012 | 51% |  | 49% | 27.5% | Admission to hospital or transfer to another hospital for admission | Patients undergoing cardiopulmonary resuscitation (CPR) or triage category ‘red’ (needing immediate care); Died in the ED; Left without being evaluated; patients attending for a planned re-evaluation. | Logistic regression |
| Marlais et al., (2011) | Acute bronchiolitis - Paediatric emergency department in London, UK. | 449 | Infants (under the age of 12 months) | April 2009 to March 2010 |  |  |  | 36.0% | Admission to hospital | Variables where missing data were more than 20% of the final data set. | Logistic regression |
| Parker et al., (2019) | Large urban acute tertiary care hospital, Singapore. | 1,232,016 | >21 Years | 1 January 2005 to 31 December 2014 | 70% |  | 30% | 38.7% | Physician decision to admit to any inpatient discipline | Dead upon arrival; Refused admission; Absconded. | Logistic regression |
| Patel et al., (2018) | Asthma - two urban paediatric EDs affiliated with one children’s hospital in the US | 29,392 | 2 - 18 years | 1 January 2012, to 31 December 2015 | 80% | 20% |  | 16.9% | Admission to hospital | Patients who received diphenhydramine; Observations with missing data. | Decision tree; Lasso logistic regression; Random forest; Gradient boosting machines |
| Peck et al., (2012) | VA federal tertiary care referral hospital, Boston, US. | 5,801 | 10 - 90+ years | 1 January to 31 May 2010 and 1-21 September 2010 | 72% (1 January to 6 May 2010) |  | 28% (7 May to 31 May 2010, and 1-12 September 2010) |  | Admission to hospital |  | Naïve Bayes; Logistic regression |
| Peck et al., (2013) | Two VHA Medical Centres, a public hospital, and a private hospital in the US. | 29,775 | 0 - 90+ years | Various dates from 17 January 2007 to 30 November 2011 for each of the 4 sites | 53.2% 58.3% 61.2% 58.3% | 26.3% 18.1% 17.4% 24.0% | 20.5% 23.6% 21.4% 16.7% | 32%, 28%, 26%, 28% | Admission to hospital |  | Logistic regression |
| Rendell et al., (2019) | Level 5 or 6 (tertiary) hospitals in the state of New South Wales, Australia | 1,721,294 | >16 years | 2013 and 2014 |  |  |  | 40.7% | Admission to short-stay and medical assessment units and transfer to another hospital. | Missing triage or uninterpretable presenting problems; Dead on arrival; Planned representations; Patients who did not wait for triage and missing or uninterpretable presenting problem field entries. | Bayesian networks; Decision tree; Logistic regression; Naïve Bayes; Neural networks; Nearest neighbour. |
| Sun et al., (2011) | Tertiary care hospital in Singapore. | 317,581 | 0 - 85+ years | 2007 & 2008 | 60% |  | 40% | 30.2% | Admission to the general ward | Died in the ED; Directly admitted to an intensive care or high dependency unit | Logistic regression |

Abbreviation: AUC, Area Under the Curve.
